# Definitive Intensity-Modulated Chemoradiation for Anal Squamous Cell Carcinoma: Outcomes and Toxicity of 428 Patients Treated at a Single Institution

**DOI:** 10.1093/oncolo/oyab006

**Published:** 2022-01-28

**Authors:** Emma B Holliday, Van K Morris, Benny Johnson, Cathy Eng, Ethan B Ludmir, Prajnan Das, Bruce D Minsky, Cullen Taniguchi, Grace L Smith, Eugene J Koay, Albert C Koong, Marc E Delclos, John M Skibber, Miguel A Rodriguez-Bigas, Y Nancy You, Brian K Bednarski, Mathew M Tillman, George J Chang, Kristofer Jennings, Craig A Messick

**Affiliations:** 1 The University of Texas MD Anderson Cancer Center, Houston, TX, USA; 2 Vanderbilt Ingram Cancer Center, Nashville, TN, USA

**Keywords:** anal squamous cell carcinoma, cisplatin, radiation dose, intensity-modulated radiation therapy, colostomy, toxicity

## Abstract

**Background:**

Although intensity-modulated radiation therapy (IMRT) is considered the standard of care for the treatment of squamous cell carcinoma of the anus (SCCA), few large series have reported oncologic outcomes and toxicities. In this retrospective report, we aim to describe outcomes and toxicities after IMRT-based chemoradiation (CRT) for the treatment of SCCA, evaluate the impact of dose escalation (>54 Gy), and compare concurrent fluoropyrimidine in combination with either mitomycin or with cisplatin as chemosensitizers.

**Methods:**

Patients treated at The University of Texas MD Anderson Cancer Center between January 1, 2003 and December 31, 2018 with IMRT-based CRT were included. Median time to locoregional recurrence, time to colostomy, and overall survival were estimated using the Kaplan–Meier method.

**Results:**

A total of 428 patients were included; median follow-up was 4.4 years. Three hundred and thirty-four patients (78.0%) were treated with concurrent cisplatin and fluoropyrimidine, and 160 (37.4%) with >54 Gy. Two- and 5-year freedom from locoregional failure, freedom from colostomy failure, and overall survival were 86.5% and 81.2%, respectively, 90.0% and 88.3%, respectively, and 93.6% and 85.8%, respectively. Neither dose escalation nor mitomycin-based concurrent chemotherapy resulted in improved outcomes. Mitomycin-based concurrent chemotherapy was associated with in approximately 2.5 times increased grade 3 or greater acute toxicity. Radiation dose >54 Gy was associated with approximately 2.6 times increased Grade 3 or greater chronic toxicity.

**Conclusions:**

Our results suggest IMRT-based CRT with concurrent fluoropyrimidine and cisplatin is a safe and feasible option for patient with SCCA and may cause less acute toxicity. The role for radiation dose escalation is unclear and requires further study.

## Teaser

Although rare, the incidence of squamous cell carcinoma of the anus continues to rise. This retrospective report describes outcomes and toxicities after intensity-modulated radiation therapy-based chemoradiation for the treatment of squamous cell carcinoma of the anus, evaluates the impact of dose escalation, and compares concurrent fluoropyrimidine in combination with either mitomycin or with cisplatin as chemosensitizers.

**Implications for Practice**

Intensity-modulated radiation with concurrent fluoropyrimidine and mitomycin is the standard of care for anal squamous cell carcinoma, but most large studies reporting oncologic outcomes are from the era of three-dimensional radiation planning. This retrospective review provides locoregional failure, colostomy failure, and overall survival rates for 428 patients treated in the modern era. These data also suggest concurrent weekly fluoropyrimidine and cisplatin may yield equivalent oncologic outcomes and potentially improved acute toxicity. Radiation dose escalation above 54 Gy did not improve outcomes in this cohort but was associated with increased chronic toxicity.

## Introduction

Although rare, the incidence of squamous cell carcinoma of the anus (SCCA) has continued to rise over the past 20 years.^1,2^ Definitive chemoradiation (CRT) is the standard of care due to high cure rates established by two randomized trials, Radiation Therapy Oncology Group (RTOG) 9811 and UK ACT II.^3,4^ Cure rates were high overall, but patients with primary tumors >5 cm or locoregional lymph node involvement at presentation had higher rates of locoregional failure (LRF).^5^ In ACT II, no statistically significant differences in survival outcomes for patients with locally advanced anal cancer were noted between cisplatin/5-fluorouracil (5FU) and mitomycin C/5FU when used as chemosensitizers with concurrent radiation.

Both RTOG 9811 and UK ACT II utilized 2D/3D conformal radiation techniques, which result in high doses to adjacent normal tissue and significant toxicity.^6,7^ The preferred radiation technique shifted to intensity-modulated radiation therapy (IMRT) after the publication of RTOG 0529, which showed IMRT resulted in reduced acute toxicity compared with patients treated with 2D and 3D conformal techniques on RTOG 9811. Additionally, patients treated with IMRT had fewer radiation treatment breaks. Oncologic outcomes were not reported in the RTOG 0529 publication,^8^ but a retrospective study of 43 patients using a similar IMRT technique reported 95% 2-year local control, 92% distant metastasis-free survival, 90% colostomy-free survival, and 94% 2-year overall survival (OS).^9^

Existing publications on IMRT-based CRT for SCCA are small and/or have limited follow-up.^10-14^ Most include a radiation dose of 50-54 Gy and concurrent mitomycin-C (MMC)/5FU as a chemosensitizer. Our aims are to (1) report LRF, colostomy failure (CF), OS, and toxicity data for patients treated with IMRT-based CRT at our institution, (2) evaluate any potential impact of dose escalation (>54 Gy), and (3) evaluate any differences between MMC-based versus cisplatin-based concurrent chemotherapy regimens.

## Methods

We obtained a waiver of consent and approval from the institutional review board for this study. All consecutive patients treated at our institution from January 1, 2003 until December 31, 2018 with IMRT-based definitive CRT for non-metastatic SCCA were included.

### Treatment Details

All patients received definitive CRT using an IMRT technique that has been described elsewhere.^13^ The primary tumor dose and fractionation were selected based on size; 50 Gy in 25 fractions for T1 tumors, 54 Gy in 27 fractions for T2 tumors, and 58 Gy in 29 fractions for T3 and T4 tumors. The majority of patients received weekly cisplatin (20mg/m^2^ intravenously once weekly) and daily 5-FU (300 mg/m^2^/day infused continuously on days of radiation) as previously reported^15^. A minority of patients were treated with MMC (10 mg/m^2^ on days 1 and 28). Occasionally, patients received capecitabine (825 mg/m^2^ twice daily orally on days of radiation) instead of 5FU. While concurrent cisplatin and 5-FU was the preferred chemotherapy regimen by our multidisciplinary treatment group, MMC was often chosen for patients with baseline renal dysfunction, significant neuropathy or hearing loss. Additionally, patients treated at our some of our regional cancer care centers were co-managed by a radiation oncologist from our institution and a medical oncologist from outside our institution. Patients managed in this way more often received concurrent MMC.

During treatment, all patients were seen weekly for toxicity assessment. Laboratory tests, including a complete blood count with differential, were also obtained weekly. Acute toxicities were graded weekly by the attending physician according to the Common Terminology Criteria for Adverse Events version 4 (CTCAEv4) and reported in weekly treatment management notes in the medical record. After treatment, patients were seen every 3 to 6 months for 5 years. Toxicities documented up to 6 weeks postcompletion of CRT were recorded as acute toxicities. Toxicities reported thereafter were recorded as late toxicities.

### Statistical Analysis

Oncologic endpoints were defined as follows: time to LRF (time from the first day of radiation to either recurrence of disease in the anal canal and/or regional lymph nodes after complete clinical response (cCR) or biopsy-proven persistence of disease at least 6 months after completion of CRT); time to colostomy (from the first day of radiation to colostomy placement either due disease recurrence or treatment-related toxicity); and OS (time from the first day of radiation to the date of death, where applicable). Patients lost to follow-up were considered censored. From these data, median time-to-event outcomes were determined using the Kaplan–Meier method. Tests for univariate time-to-event analysis included the log-rank test and Cox proportional hazards regression. Cox regression was used for the multivariate analysis for event endpoints to assess the relationship between prognostic factors and oncologic endpoints of interest. Stepwise regression was used to determine the most informative set of variables, with the Bayesian Information Criterion as the complexity-penalizing criterion. Toxicity endpoints were assessed with multivariate and variable-selected logistic regression models. Standard dose radiation was defined as ≤54 Gy and dose-escalated radiation was defined as >54 Gy. *P*-values of < .05 were considered statistically significant. Software used for analysis was R version 4.0.5 (R Core Team, 2021).

## Results

### Patient Demographics

Four hundred twenty-eight patients were included. The median [interquartile range (IQR)] follow-up from the start of CRT was 4.4 [2.73-7.09] years. Patient characteristics by tumor stage are listed in Table 1.

**Table 1.** Patient, tumor, and treatment characteristics by T-stage.

	Total *N* = 462	T1 *N* = 80 (18.7%)	T2 192 (44.9%)	T3 105 (24.5%)	T4 51 (11.9%)	*P* ^a^
Age						
Median [IQR]	60 years [52-67]	60 years [53-67]	61 years [52-67]	59 years [52-68]	59 years [52-66]	
≤65 years	305 (71.3%)	58 (72.5%)	135 (70.3%)	74 (70.5%)	38 (74.5%)	.932
>65 years	123 (28.7%)	22 (27.5%)	57 (29.7%)	31 (29.5%)	13 (25.5%)	
Sex						
Female	318 (74.3%)	66 (82.5%)	146 (76.0%)	72 (68.6%)	34 (66.7%)	.090
Male	110 (25.7%)	14 (17.5%)	46 (24.0%)	33 (31.4%)	17 (33.3%)	
HIV status^b^						
Negative	408 (95.3%)	78 (97.5%)	182 (94.8%)	99 (94.3%)	49 (96.1%)	.731
Positive	20 (4.7%)	2 (2.5%)	10 (5.2%)	6 (5.7%)	2 (3.9%)	
Smoking history						
Never	213 (49.8%)	48 (60.0%)	103 (53.6%)	47 (44.8%)	16 (31.4%)	<.001
Former	148 (34.6%)	26 (32.5%)	68 (35.4%)	31 (29.5%)	23 (45.1%)	
Current	66 (15.4%)	6 (7.5%)	21 (10.9%)	27 (25.7%)	12 (23.5%)	
Excisional biopsy prior to RT						
No	318 (74.3%)	36 (45.0%)	142 (74.0%)	91 (86.7%)	49 (96.1%)	<.001
Yes	110 (25.7%)	44 (55.0%)	50 (26%)	14 (13.3%)	2 (3.9%)	
N-stage						
N0	210 (49.1%)	60 (75.0%	107 (55.7%)	32 (30.5%)	11 (21.6%)	<.001
N1	218 (50.9%)	20 (25.0%)	85 (44.3%)	73 (69.5%)	40 (78.4%)	
Radiation dose						
Median [IQR]	54 Gy [54-58]	50 Gy [50-50]	54 Gy [54-54]	58 Gy [58-58]	58 Gy [58-58]	
≤54 Gy	268 (62.6%)	79 (98.8%)	175 (91.1%)	10 (9.5%)	4 (7.8%)	<.001
>54 Gy	160 (37.4%)	1 (1.2%)	17 (8.9%)	95 (90.5%)	47 (92.2%)	
Concurrent chemotherapy						
Cis	334 (78.0%)	63 (78.8%)	152 (79.2%)	78 (74.3%)	41 (80.4%)	.846
MMC	73 (17.1%)	13 (16.3%)	30 (15.6%)	23 (21.9%)	7 (13.7%)	
Other^c^	21 (4.9%)	4 (5.0%)	10 (5.2%)	4 (3.8%)	3 (5.9%)	
Time from diagnosis to RT						
Median [IQR]	47d [34-62]	53d [40-66]	48d [36-62]	41d [32-53]	46d [29-85]	
≤42 days	268 (58.0%)	27 (33.8%)	76 (39.6%)	60 (57.1%)	22 (43.1%)	.007
>42 days	160 (42.0%)	53 (66.3%)	116 (60.4%)	45 (42.9%)	29 (56.9%)	
Radiation treatment break						
No	379 (88.6%)	73 (91.3%)	167 (87.0%)	93 (88.6%)	46 (90.2%)	.758
Yes	49 (11.4%)	7 (8.7%)	25 (13%)	12 (11.4%)	5 (9.8%)	

Pearson chi-square test.

Only 3 HIV+ patients had a CD4 count <200 at the time of treatment initiation.

Other chemotherapy included 5-fluorouracil monotherapy, capecitabine monotherapy or capecitabine + oxaliplatin.

Abbreviations: Cis, cisplatin; Gy, gray; HIV, human immunodeficiency virus; IQR, interquartile range; MMC, mitomycin C; RT, radiation therapy.

### Locoregional Failure

Three hundred ninety-six patients (92.5%) achieved a cCR. The median time to cCR was 2.8 [IQR 1.8-4.2] months. Fifty-seven patients (13.3%) experienced persistent or recurrent locoregional disease. Estimated 2- and 5-year freedom from LRF was 85.7% (95% CI 82.5%, 90.1%) and 79.7% (95% CI 75.7%, 83.9%), respectively. Univariate analysis is shown in Table 2. In the multivariable model, factors significantly associated with LRF included being HIV positive (HR: 3.146 (95% CI 1.501-6.595); *P* = .008), being a current smoker (HR: 2.206 (95% CI 1.272-3.825); *P* = .02) and receiving >54 Gy (HR: 3.348 (95% CI 2.076-5.399); *P* < .001).

**Table 2.** Univariate analysis of factors associated with locoregional failure, colostomy failure, and overall survival.

	*N* (%)	Locoregional failure		Colostomy failure		Overall survival	
		HR (95% CI)	*P*	HR (95% CI)	*P*	HR (95% CI)	*P*
Age							
≤65 years	305 (71.3%)	Ref		Ref		Ref	
>65 years	123 (28.7%)	0.785 (0.469-1.316)	.358	0.528 (0.246-1.131)	.101	1.219 (0.715-2.079)	.467
Sex							
Female	318 (74.3%)	Ref		Ref		Ref	
Male	110 (25.7%)	1.248 (0.768-2.029)	.371	1.068 (0.553-2.062)	.845	1.707 (1.013-2.878	.045
HIV status							
Negative	408 (95.3%)	Ref		Ref		Ref	
Positive^a^	20 (4.7%)	3.000 (1.443-6.238)	.003	2.831 (1.118-7.168)	.028	3.237 (1.471-7.122)	.004
Smoking							
Never	213 (49.8%)	Ref		Ref		Ref	
Former	148 (34.6%)	1.155 (0.676-1.973)	.599	1.163 (0.598-2.265)	.656	0.864 (0.473-1.578)	.634
Current	66 (15.4%)	2.750 (1.600-4.725)	<.001	1.703 (0.792-3.664)	.173	1.981 (1.072-3.659)	.029
Excision before RT							
No	318 (74.3%)	Ref		Ref		Ref	
Yes	110 (25.7%)	0.362 (0.181-0.726)	.004	0.503 (0.225-1.126)	.095	0.312 (0.134-0.726)	.007
T-stage							
T1	80 (18.7%)	Ref		Ref		Ref	
T2	192 (44.9%)	1.581 (0.688-3.632)	.280	1.581 (0.525-4.764)	.416	1.450 (0.588-3.581)	.420
T3	105 (24.5%	3.348 (1.466-7.645)	.004	3.004 (0.997-9.055)	.051	2.699 (1.089-6.693)	.032
T4	51 (11.9%)	4.055 (1.668-9.862)	.002	5.384 (1.736-16.702)	.004	3.305 (1.248-8.755)	.016
N-stage							
N0	210 (49.1%)	Ref		Ref		Ref	
N1	218 (50.9%)	1.94 (1.223-3.076)	.005	1.972 (1.045-3.517)	.036	1.573 (0.941-2.63)	.084
RT dose							
≤54 Gy	268 (62.6%)	Ref		Ref		Ref	
>54 Gy	160 (37.4%)	3.578 (2.247-5.698)	<.001	3.082 (1.693-5.610)	<.001	2.780 (1.664-4.695)	<.001
Concurrent Chemo							
Cisplatin	334 (78.0%)	Ref		Ref		Ref	
MMC	73 (17.1%)	0.945 (0.508-1.756)	.858	0.621 (0.244-1.578)	.317	1.641 (0.894-3.011)	.110
Other^b^	21 (4.9%)	1.889 (0.862-4.140)	.112	1.330 (0.410-4.318)	.635	1.944 (0.821-4.603)	.131
Time to RT							
Median [IQR]	47d [34-62]						
≤42 days	268 (58.0%)	Ref		Ref		Ref	
>42 days	160 (42.0%)	3.579 [2.247-5.698]	<.001	3.082 [1.693-5.610]	<.001	2.795 [1.664-4.695]	<.001
RT break							
No	379 (88.6%)	Ref		Ref		Ref	
Yes	49 (11.4%)	1.337 (0.688-2.597)	.392	1.344 (0.570-3.175)	.499	2.451 (1.340-4.464)	.004

Only 3 HIV+ patients had a CD4 count <200 at the time of treatment initiation.

Other chemotherapy included 5-fluorouracil monotherapy, capecitabine monotherapy or capecitabine + oxaliplatin.

Abbreviations: CI, confidence interval; Cis, cisplatin; Gy, gray; HIV, human immunodeficiency virus; HR, hazard ratio; IQR, interquartile range; MMC, mitomycin C; Ref, reference; RT, radiation therapy.

### Colostomy Failure

Seven patients required a diverting colostomy prior to treatment initiation due to fistula, obstruction, or pain. Forty-seven patients (11.0%) had a colostomy at last follow-up, either for recurrent or persistent disease (*N* = 39) or for the management of side effects of radiation (*N* = 8). Estimated 2- and 5-year freedom from colostomy were 90.0% and 88.3%, respectively. Univariate analysis is shown in Table 2. In the multivariable model, only receiving >54 Gy (HR 3.082 (95% CI 1.693-5.610); *P* < .001) was associated with CF.

### Overall Survival

Three hundred sixty-four patients (85.0%) were alive at last follow-up. Estimated 2- and 5-year OS were 93.6% and 85.8%, respectively. Univariate analysis is shown in Table 2. In the multivariable model factors associated with worse OS included being HIV positive (HR: 2.884 (95% CI 1.295-6.419); *P* = .022), receiving >54 Gy (HR: 2.411 (1.474-4.978); *P* < .001) and having an unplanned treatment break (HR: 2.709 (95% CI 1.474-4.978); *P* = .003). Having an excision prior to CRT was associated with improved OS (HR: 0.358 (95% CI 0.149-0.859); *P* = .010).

### Impact of T-Stage on Oncologic Outcomes

**Figure 1.**
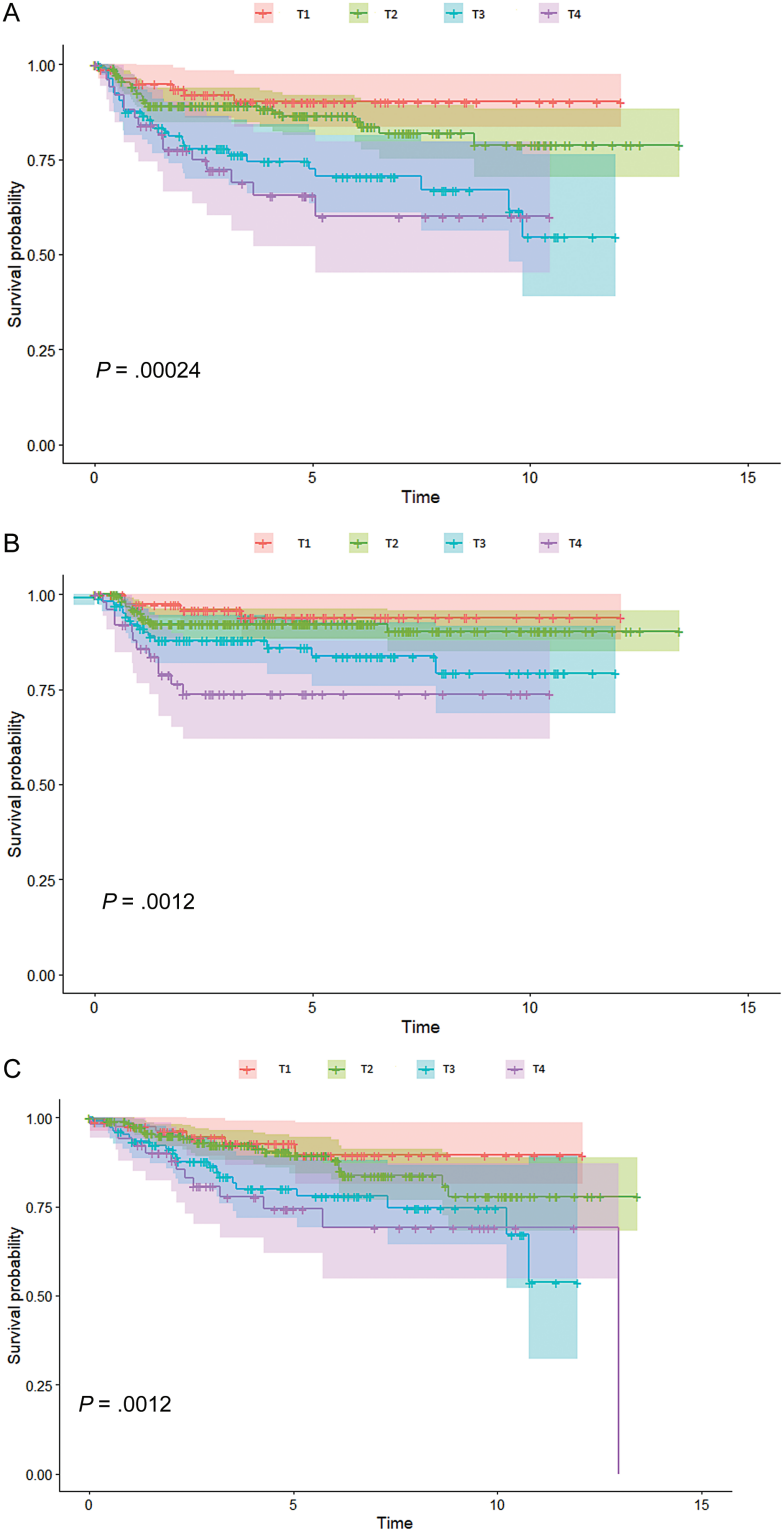
The freedom from locoregional failure (A), freedom from colostomy (B), and overall survival (C) for patients with anal cancer treated with IMRT-based chemoradiation stratified by T-stage.

When radiation dose was removed from the multivariable model, increasing T-stage was associated with worse LRF (HR with each increasing stage 1.698 (95% CI 1.361, 2.12); *P* < .001), CF (HR per stage increase 1.668 (95% CI 1.248, 2.231); *P* < .001), and OS (HR by stage: 1.567 (95% CI 1.204, 2.040); *P* < .001). Locoregional failure, CF, and OS of patients with T1, T2, T3, and T4 tumors are presented in Figure 1A, B, and C, respectively.

### Toxicity

Forty-nine patients (11.4%) required an unplanned radiation treatment break of ≥1 day (median 3 days, IQR [2-7]), and 63 patients (14.7%) required hospitalization during treatment. One hundred thirty-nine patients (32.5%) developed ≥1 acute G3+ gastrointestinal (*N* = 56), genitourinary (*N* = 11), or dermatologic toxicity (*N* = 90). Six patients experienced G4, and one patient experienced G5 acute gastrointestinal toxicity. In the multivariable model, MMC remained significantly associated with increased acute G3+ gastrointestinal, genitourinary, and dermatologic toxicity (OR: 2.484 (95% CI 1.480-4.175); *P* < .001; Table 3). Patients receiving concurrent MMC were more likely to have G3+ neutropenia (30.9% vs 3.5%; *P* < .001; Table 4). Thirty-nine patients (9.1%) developed at least one chronic G3+ gastrointestinal (*N* = 32), genitourinary (*N* = 39), or dermatologic toxicity (*N* = 1). Most were G3 toxicities, but there were nine patients who experienced G4 gastrointestinal toxicity and two patients who experienced G4 genitourinary toxicity. In the multivariable model, receiving >54 Gy was the only factor significantly associated with increased late toxicity (OR: 2.644 (95% CI 1.361-5.257); *P* = .005; Table 3).

**Table 3.** Univariate analysis of factors associated with acute and late Grade 3 or higher gastrointestinal, genitourinary, and dermatology toxicity.

	*N* (%)	Acute G3+ GI, GU and dermatologic toxicity; *N* = 139 patients			Late G3+ GI, GU and dermatologic toxicity *N* = 39 patients		
		*N* (row %)	OR (95% CI)	*P*	*N* (%)	OR (95% CI)	*P*
Age							
≤65 years	305 (71.3%)	92 (30.2%)	Ref		26 (8.5%)	Ref	
>65 years	123 (28.7%)	77 (62.6%)	1.431 (0.899-2.267)	.118	13 (10.6%)	1.267 (0.576-2.667)	.101
Sex							
Female	318 (74.3%)	98 (30.8%)	Ref		25 (7.9%)	Ref	
Male	110 (25.7%)	41 (37.3%)	1.333 (0.822-2.147	.238	14 (12.7%)	1.707 (0.787-3.574)	.129
HIV status							
Negative	408 (95.3%)	134 (32.8%)	Ref		37 (9.1%)	Ref	
Positive^a^	20 (4.7%)	5 (25%)	0.682 (0.190-2.028)	.626	2 (10.0%)	2.831 (1.118-7.168)	.028
Smoking							
Never	213 (49.8%)	59 (27.7%)	Ref		15 (7.0%)	Ref	
Former	148 (34.6%)	56 (37.8%)	1.587 (0.990-2.547)	.051	16 (10.8%)	1.598 (0.713-3.601)	.252
Current	66 (15.4%)	24 (36.4%)	1.489 (0.790-2.771)	.217	7 (10.6%)	1.563 (0.514-4.310)	.432
Excision before RT							
No	318 (74.3%)	113 (35.5%)	Ref		31 (9.7%)	Ref	
Yes	110 (25.7%)	26 (23.6%)	0.562 (0.328-0.942)	.025	8 (7.3%)	0.727 (0.279-1.684)	.565
T-stage							
T1	80 (18.7%)	19 (23.8%)	Ref		6 (7.5%)	Ref	
T2	192 (44.9%)	60 (31.3%)	1.457 (0.777-2.819)	.243	10 (5.5%)	0.679 (0.214-2.358)	.572
T3	105 (24.5%	39 (37.1%)	1.891 (0.949-3.861)	.057	11 (10.5%)	1.440 (0.463-4.975)	.610
T4	51 (11.9%)	21 (41.2%)	2.233 (0.980-5.144)	.051	12 (23.5%)	3.754 (1.194-13.18)	.017
N-stage							
N0	210 (49.1%)	67 (31.9%)	Ref		20 (9.5%)	Ref	
N1	218 (50.9%)	72 (33.0%)	1.052 (0.688-1.611)	.837	19 (8.7%)	0.907 (0.443-1.853)	.867
RT dose							
≤54 Gy	268 (62.6%)	78 (29.1%)	Ref		16 (6.0%)	Ref	
>54 Gy	160 (37.4%)	61 (38.1%)	1.499 (0.970-2.316)	.056	23 (14.4%)	2.638 (1.285-5.537)	.005
Concurrent Chemo							
Cisplatin	334 (78.0%)	94 (28.1%)	Ref		31 (9.3%)	Ref	
MMC	73 (17.1%)	36 (49.3%)	2.478 (1.429-4.301)		6 (8.2%)	0.876 (0.287-2.246)	1.00
Other^b^	21 (4.9%)	9 (75%)	1.911 (0.687-5.129)	<.001 .213	2 (9.5%)	1.029 (0.111-4.604)	1.00
Time to RT							
≤42 days	268 (58.0%)	57 (21.3%)	Ref		21 (7.8%)	Ref	
>42 days	160 (42.0%)	82 (51.3%)	3.579 [2.247-5.698]	<.001	18 (11.3%)	0.626 (0.304-1.277)	.177

Only 3 HIV+ patients had a CD4 count <200 at the time of treatment initiation.

Other chemotherapy included 5-fluorouracil monotherapy, capecitabine monotherapy or capecitabine + oxaliplatin.

Abbreviations: CI, confidence interval; Cis, cisplatin; Gy, gray; HIV, human immunodeficiency virus; HR, hazard ratio; IQR, interquartile range; MMC, mitomycin C; Ref, reference; RT, radiation therapy.

**Table 4.** Treatment-related toxicity per concurrent chemotherapy regimen and radiation dose.

*N* (%)	Total (*N* = 428)	Concurrent chemotherapy				Radiation dose		
		Cisplatin-containing (N = 334)	MMC-containing (N = 73)	Other (*N* = 21)^a^	*P* ^b^	≤54 Gy (*N* = 268)	>54 Gy (*N* = 160)	*P* ^b^
Acute Grade 3+ neutropenia^c^								
Yes	28 (7.0%)	11 (3.5%)	17 (30.9%)	0 (0%)		13 (5.2%)	15 (10.0%)	
No	373 (93.0%)	317 (96.5%)	38 (69.1%)	18 (100%)	<.001	238 (94.8%)	135 (90.0%)	.067
Acute Grade 3+ GI, GU or skin toxicity								
Yes	139 (32.5%)	94 (28.1%)	36 (49.3%)	9 (42.9%)		83 (31.0%)	56 (35.0%)	
No	289 (67.5%)	240 (71.9%)	37 (50.7%)	12 (57.1%)	.001	185 (69.0%)	104 (65.0%)	.389
Unplanned RT treatment break								
Yes	49 (11.4%)	30 (9.0%)	15 (20.6%)	4 (19.1%)	.010	25 (9.3%)	24 (15%)	
No	379 (88.6%)	304 (91.0%)	58 (79.5%)	17 (80.9%)		243 (90.7%)	136 (85%)	.075
Hospitalization during treatment								
Yes	63 (14.7%)	39 (11.7%)	21 (29.2%)	3 (14.3%)		40 (15.0%)	23 (14.4%)	
No	364 (85.3%)	295 (88.3%)	51 (70.8%)	18 (85.7%)	<.001	227 (85.0%)	137 (85.6%)	.864
Late Grade 3+ GI, GU or skin toxicity								
Yes	39 (9.1%)	31 (9.3%)	6 (8.2%)	2 (9.5%)		26 (9.7%)	13 (8.1%)	
No	389 (90.9%)	303 (90.7%)	67 (91.8%)	19 (90.5%)	.958	242 (90.3%)	147 (91.9%)	.584

Other chemotherapy included 5-fluorouracil monotherapy, capecitabine monotherapy or capecitabine + oxaliplatin.

Pearson chi-square test.

Laboratory values available for *N* = 401 patients.

Abbreviations: GI, gastrointestinal; GU, genitourinary; Gy, gray; MMC, mitomycin C.

## Discussion

In this retrospective analysis of 428 consecutive SCCA patients treated with IMRT-based CRT, over 90% achieved a cCR after CRT; at 5 years, freedom from LRF was 80%, freedom from colostomy was 88% and OS was 86%. Our outcomes compare favorably with cooperative group trials completed in the 3-D era. Patients treated on RTOG 9811 had a 5-year disease-free survival (DFS) of 68% in the MMC/5FU arm and 58% in the cis/5FU arm. 5-year OS was 78% vs 71%, respectively^4^. Approximately 90% of patients treated on UK ACT II experienced a cCR at 26 weeks, and 3-year progression-free survival (PFS) was approximately 74%^3^. Although concurrent MMC/5FU remains the standard of care^16^, our institution has favored using weekly low dose cisplatin and daily 5FU concurrent with radiation^15^. Although retrospective results are no substitute for a randomized trial to establish the standard of care, we are reassured that our outcomes are similar to smaller reports outlining outcomes after IMRT^10-14^.

T-stage is established as a prognostic factor for LRF and DFS^5,6,17^. Interestingly, radiation dose >54 Gy was a stronger predictor than T-stage for worse LRF, CF, and OS in our analysis. T-stage is colinear with dose in our cohort given our practice of prescribing 50 Gy for T1, 54 Gy for T2, and 58 Gy for T3-T4 tumors. After removing radiation dose from the multivariate models, T-stage was significantly associated with worse outcomes. That dose demonstrated a greater strength of correlation with adverse outcomes than T-stage suggests that residual confounding likely impacted the dose variable. In our practice, there is some variation in radiation dose selection for individual patients. For example, larger T2 tumors with other adverse features may have received >54 Gy, while small (eg, just exceeding 5 cm), more favorable T3/T4 tumors may have received ≤54 Gy. Additionally, patients who received partial excisional may have received radiation doses based on their T-stage at presentation rather than postexcision.

Although we do not believe radiation >54 Gy directly causes worse LRF, CF, and OS, in our study it was also not associated with improved outcomes. Ours is not the first study demonstrating a lack of obvious benefit for dose escalation for advanced tumors^11,18^. We cannot make a clear comment on the benefit of dose escalation based on our data and await the results from PLATO ACT5 which is currently evaluating dose escalation for locally advanced tumors either to 61.6 or 58.5 Gy compared with 53.2 Gy^19^. Additionally, the Eastern Cooperative Oncology Group (ECOG) EA2165 study is evaluating adjuvant nivolumab after CRT as a means of treatment escalation for patients with locally advanced disease^20^.

We found current smoking, living with HIV and receiving an unplanned CRT break were associated with worse oncologic outcomes. Others have also demonstrated the association of smoking with worse LRF and OS^21^. Although studies in the pre-antiretroviral therapy era suggest worse mortality and morbidity for patients living with HIV^22^, modern data have shown comparable outcomes to patients without HIV^23^. Fewer than 5% in our series were living with HIV, which may have influenced our results. Database studies have shown the relationship with prolongation of CRT and worse survival^24,25^. The finding that pre-CRT excision was associated with improved OS is likely due to the fact that receipt of excision is associated with early-stage disease without prognostic significance on its own. Indeed, over 85% of patients who had excision had T1-T2 disease. Although beyond the scope of this manuscript, we acknowledge the controversy regarding the role of local excision in the management of patients with very early stage SCCA^26^.

RTOG 0529 showed lower rates of G3+ acute genitourinary (2%), dermatologic (23%), and gastrointestinal toxicities (21%) in patients treated with IMRT compared with patients treated with conformal radiation on RTOG 9811. In our series, G3+ acute genitourinary and dermatologic toxicities were similar at 2.6% and 21.3%, while rates of G3+ acute gastrointestinal toxicities were lower at 13%. Receiving MMC-containing concurrent chemotherapy was associated with worse acute G3+ non-hematologic toxicities, G3+ neutropenia, unplanned breaks in radiation and hospitalizations. The only factor significantly associated with increased late G3+ non-hematologic toxicities was receipt of >54 Gy. Prospectively collected patient-reported-outcomes are needed for optimal assessment of the toxicity profile, however. Ongoing cooperative group trials are evaluating treatment de-escalation with reduced radiation dose and/or volume as a means of reducing toxicity.^19,27^

Limitations of this study include a lack of prospective toxicity collection throughout the duration of the study as well as a lack of patient-reported outcomes. There may also be selection bias of those able to receive treatment at a tertiary cancer center which may lead to improved outcomes. Despite these limitations, this report adds meaningfully to the existing literature, representing the largest modern cohort of patients treated with definitive IMRT-based CRT. Furthermore, unlike previously published studies, most patients in our cohort were treated with concurrent cisplatin/5FU without loss of treatment efficacy.

In conclusion, patients in this retrospective study treated with IMRT and weekly low dose cisplatin and daily 5FU had similar outcomes to those treated with MMC and 5FU but with less acute toxicity, fewer hospitalizations and fewer radiation treatment breaks. Prospective data are needed to validate these observations. In this study, dose escalation >54 Gy did not yield superior outcomes and resulted in increased chronic toxicity. Further study is needed to evaluate whether selective dose escalation may have a benefit patients at increased risk for local recurrence.

## Data Availability

The data underlying this article will be shared on reasonable request to the corresponding author.
